# Complement and inflammasome crosstalk in chronic inflammation

**DOI:** 10.3389/fimmu.2026.1778759

**Published:** 2026-03-30

**Authors:** Larisa Janžič, Katarina Kouter

**Affiliations:** Institute of microbiology and immunology, Faculty of medicine, University of Ljubljana, Ljubljana, Slovenia

**Keywords:** chronic inflammation, complement system, inflammasomes, innate immunity, NLRP3

## Abstract

Chronic inflammation underlies a broad range of human diseases, including autoimmune disorders, neurodegeneration, and metabolic syndromes. While acute inflammation is essential for pathogen clearance and tissue repair, persistent activation leads to tissue damage and disease progression. Two key innate immune pathways, the complement system and inflammasomes, are crucial mediators of inflammation and are increasingly recognized as interdependent effectors that sustain chronic inflammatory states. This review examines the mechanistic crosstalk between complement activation and inflammasome signaling, with an emphasis on the NLRP3 inflammasome. We first outline how complement pathways drive inflammation through cell recruitment, cytokine induction, and failure of regulatory checkpoints. Next, we review the triggers, regulation, and persistence of inflammasome activation, highlighting the central role of NLRP3 and its engagement by diverse danger signals in chronic disease. In the main section, we detail multiple mechanistic intersections between the two systems, including shared activation triggers such as reactive oxygen species and mitochondrial damage, direct priming and activation of inflammasomes by complement components (e.g., C3a, C5a, MAC), and feedback loops driven by inflammasome-derived cytokines (IL-1β, IL-18) that enhance complement activity and immune cell recruitment. We further illustrate these interactions across disease contexts, including gout, atherosclerosis, rheumatoid arthritis, systemic lupus erythematosus, and Alzheimer’s disease. In each case, complement and inflammasomes form a self-amplifying loop that exacerbates inflammation and tissue damage. We also examine the dual role of C1q as both an enhancer and suppressor of inflammasome activation, depending on the cellular and molecular environment. Finally, we discuss therapeutic strategies targeting these pathways. Complement inhibitors (e.g., eculizumab, avacopan), inflammasome inhibitors (e.g., MCC950), and IL-1β blockers (anakinra) show clinical promise, and dual-targeting approaches may offer synergistic benefit. Understanding the interplay between complement and inflammasomes provides critical insight into the persistence of inflammation and opens new avenues for precise immunomodulation in chronic diseases.

## 1 Introduction

Chronic inflammation, generally defined as a slow, long-term inflammatory state lasting months or years, underlies a wide range of human diseases, including autoimmune disorders, cardiovascular conditions, neurodegeneration, and metabolic syndromes. Unlike acute inflammation, which resolves after pathogen clearance or tissue repair, chronic inflammation persists due to sustained activation of immune pathways. This persistent activation is often sterile, driven not by microbes but by endogenous danger signals, and is characterized by unresolved cytokine production, tissue remodeling, and cellular stress (1, 2). Understanding the molecular circuits that maintain such non-resolving inflammation is critical for designing effective therapeutic strategies.

Among the innate immune components implicated in chronic inflammation, the complement system and the inflammasome complex stand out as central effectors. The complement cascade, classically associated with microbial defense and immune complex clearance, also acts as a potent amplifier of inflammation through its cleavage products C3a and C5a and the membrane attack complex (MAC). Similarly, inflammasomes – particularly the nucleotide-binding oligomerization domain (NOD)-, leucine-rich repeat (LRR)-, and pyrin domain (PYD)-containing protein 3 (NLRP3) inflammasome – are cytosolic multiprotein platforms that sense diverse danger signals and activate caspase-1 to process proinflammatory cytokines such as interleukin-1β (IL-1β) and interleukin-18 (IL-18). These cytokines not only orchestrate local inflammation but also create feedback loops that perpetuate their own production and tissue recruitment (3, 4).

Emerging evidence shows that the complement and inflammasome systems are not isolated but are intricately connected. Complement-derived signals such as MAC, C3a, and C5a can serve as either “Signal 1” (priming) or “Signal 2” (activation) for the inflammasome, particularly the NLRP3 inflammasome (3). Inflammasome-activated caspase-1 processes proinflammatory cytokines such as IL-1β and IL-18 into their active forms, which then stimulate the production of other inflammatory mediators (e.g., IL-6, TNF-α, chemokines) and promote an inflammatory form of cell death called pyroptosis, thereby amplifying the initial inflammatory response (5). During an inflammatory flare (e.g., in sepsis or arthritis), IL-1β and other cytokines can increase complement synthesis, ensuring abundant substrate for ongoing complement activation. This creates an inflammatory feed-forward loop in which complement activation products promote inflammasome-driven cytokine release, and those cytokines, in turn, increase local complement synthesis, fueling further inflammation and tissue damage (6–8).These interactions are context-dependent and contribute to inflammation that is both robust and resistant to resolution. Understanding this crosstalk provides new insight into the mechanisms of disease persistence and offers avenues for therapeutic intervention.

## 2 The complement system

The complement is a complex and highly regulated cascade system, comprised of over 30 different components. Evolutionary speaking, it is an ancient evolutionary acquisition, observed as early as in Porifera (sea sponges) (9). The complement system serves as one of the key innate effectors, that in turn also serves as bridge between innate and adaptive immune responses (10). It plays a major role in host defense against pathogens, where activation of the cascade ends in the formation of a pore-forming structure that lyses susceptible target cells. In parallel, complement activation leads to surface deposition of complement fragments that act as opsonins, enhancing phagocytosis. Complement-derived peptides also promote immune cell recruitment, inflammation, and activation of platelets and epithelial cells. Beyond immune defense, the complement system plays a critical role in tissue maintenance by facilitating immune clearance of cellular debris and apoptotic material (11). Importantly, complement activity is also involved in synaptic pruning, a key neurodevelopmental process in which inactive or weak synapses are eliminated to refine neuronal circuits (12).

### 2.1 Complement activation

The complement system can be activated through three main pathways: the classical, the lectin and the alternative; with the major difference being the activation trigger and the formation of convertase complexes.

Classical pathway is triggered by immune complexes containing IgG or IgM antibodies. These complexes are recognized by C1q, which binds to the Fc region of antibodies or directly to certain surfaces. Binding of C1q induces conformational changes that activate the associated serine proteases C1r and C1s. Activated C1s cleaves C4 into C4a and C4b, with C4b covalently attaching to the target surface. Surface-bound C4b then binds C2, which is subsequently cleaved by C1s into two fragments, a larger (now commonly known as C2b) and smaller (historically named C2b, but with recent nomenclature suggestion, renamed to C2a) (13, 14). As a result, the C3 convertase of the classical pathway is now referred to as C4b2b, though older literature may still use the designation C4b2a (14). This C3 convertase cleaves C3 into C3a and C3b. Incorporation of C3b into the convertase forms the C5 convertase (C4b2b3b), which cleaves C5 into the proinflammatory peptide C5a and the MAC-initiating fragment C5b (15).

Lectin pathway bears a close resemblance to the classical pathway. Both classical and lectin pathways are believed to evolutionary develop simultaneously and first appeared in *Coelenterata* (*Cnidaria* and *Ctenophora*) (9). Rather than antibodies, the lectin pathway is initiated by pattern recognition molecules (PRMs), such as mannose-binding lectin (MBL), ficolins, and collectins. These PRMs recognize specific carbohydrate patterns present on microbial surfaces or altered host cells. After binding to a target surface, PRMs associate with MBL-associated serine proteases (MASPs). MASP-1 undergoes autoactivation and subsequently activates MASP-2. While MASP-1 can cleave C2, MASP-2 is capable of cleaving both C4 and C2, leading to formation of the classical/lectin pathway C3 convertase C4b2b and continuation of the cascade (16).

Evolutionary speaking, the alternative pathway is the oldest of the three complement pathways (9). It is characterized by low-level, continuous spontaneous activation through hydrolysis of C3 to C3(H_2_O), a process known as the “tickover” mechanism (17). This basal activity allows the alternative pathway to function as a surveillance system, rapidly responding to foreign or altered surfaces. C3(H_2_O) and surface-bound C3b can bind factor B, which is subsequently cleaved by factor D into fragments Ba and Bb. Bb remains associated with C3(H_2_O) or C3b, forming the fluid-phase C3 convertase C3(H_2_O)Bb or the surface-bound alternative pathway C3 convertase C3bBb. Because this convertase is unstable, it requires stabilization by properdin, which enhances convertase longevity and activity. Addition of another C3b molecule to C3bBb generates the alternative pathway C5 convertase, C3bBbC3b (18).

Although complement activation is conceptually described as proceeding through three distinct pathways, accumulating evidence demonstrates cross-activation and interplay of the three pathways. Such example is the amplification loop, a defining feature of the alternative pathway. C3b, generated by cleavage of C3, can be produced through activation of any of the three pathways. The alternative pathway amplification loop continuously amplifies complement activation by using deposited C3b to form the C3bBb convertase, which generates additional C3b regardless of the initiating pathway (19). This initiates additional rounds of pathway activation, resulting in a very fast and robust production of C3b. Such mechanism allows for the interplay of both classical and lectin pathway with the alternative pathway, as C3b generated by classical or lectin pathways stabilizes and accelerates alternative pathway activation. This amplification mechanism allows for rapid opsonization and immune activation but also necessitates strict regulatory control (11). Beyond convertase-driven amplification, soluble PRMs such as pentraxins provide an additional level of pathway cross-activation at the recognition stage. Pentraxins are a protein superfamily that includes C-reactive protein (CRP), serum amyloid P component (SAP) and pentraxin 3 (PTX3). They are classified as the acute phase proteins, meaning their concentration is associated with inflammatory states and tissue damage. Pentraxins possess immunoglobulin-like similarities, acting as an ancient precursor to antibodies (20). Pentraxins can initiate activation of both the classical and lectin pathways. Pentraxins bound to a target surface recruit C1q, triggering activation of the C1r–C1s complex and subsequent cleavage of C4 and C2, leading to formation of the classical pathway C3 convertase (C4b2b). Surface-bound pentraxins can also interact with lectin pathway recognition molecules such as MBL and ficolins, promoting cleavage of C4 and C2 and generation of the same C3 convertase (C4b2b). Because both C1q and lectin pathway recognition molecules–MASP complexes can assemble on surfaces with bound pentraxin, heterocomplexes can form. This cross-activation of the classical and lectin pathway can occur simultaneously and reinforce each other locally (21). The resulting C3b, generated downstream of classical or lectin pathway activation subsequently feeds into the alternative pathway amplification loop, linking all three pathways. In addition to initiating classical and lectin pathway activation, pentraxins can interact with complement regulators such as factor H and C4BP, shaping where amplification is allowed or restrained (22).

Activation of any of the three complement pathways can lead to the terminal pathway, which is initiated by the cleavage of C5 by C5 convertases originating from the classical, lectin, or alternative pathways. The resulting C5a fragment acts as a potent anaphylatoxin and chemoattractant, while C5b triggers the assembly of the terminal complement complex (TCC, MAC, or C5b-9). C5b rapidly binds to C6 and C7 to form the C5b-6-7 complex, which binds to the target membrane. Further binding of C8 induces conformational changes that allow partial insertion into the membrane. Finally, multiple C9 molecules polymerize to form a transmembrane pore, leading to osmotic lysis of the target cell (23). MAC-mediated lysis is particularly important for defense against certain bacterial pathogens, and individuals with inherited deficiencies of terminal complement components are at increased risk of infections, particularly with *Neisseria meningitidis* and *Streptococcus pneumoniae* (24).

### 2.2 Complement regulation

The complement system requires tight regulation to prevent damage to host tissue and maintain effective immune defense. Excessive or inappropriate activation, for example on biomaterials or after transplantation, can damage self-cells, while deficiency or insufficient complement activation impairs the immune response and promotes autoimmune or chronic inflammatory diseases (25, 26). Regulation is mediated by soluble and membrane-bound inhibitors that prevent excessive activation, host tissue damage, and persistent inflammatory signaling. C1 inhibitor (C1-INH), factor I, and C4b-binding protein (C4BP) primarily control the classical and lectin pathways, while factor H and factor I regulate the alternative pathway (26, 27). C1-INH inhibits C1r/C1 and MASP and also modulates the contact system. Factor I inactivates C3b and C4b with cofactors including factor H, C4BP, MCP, and CR1. In addition, decay-promoting factor (DAF) limits complement activation by destabilizing C3 convertases (16). Additionally, mechanisms are in place to prevent MAC from self-membrane perforation, such as CD59, clusterin and vitronectin (inhibiting membrane attack complex assembly or insertion). For a comprehensive overview of the complement pathways regulation please refer to Merle et al. (2015) (18).

### 2.3 Complosome

In recent years, research has expanded on the additional role and functions of the complement system. The term complosome has been presented to describe the intracellular activity of the complement system, as opposed to the well-known and described extracellular activity. Compared to the anti-pathogen antigenic activity of serum active complement system, complosome appears to be involved in normal, both immune and non-immune cell physiology processes, such as cell metabolism, autophagy, vesicular transport, and gene expression and protein translation (28). The key players of the composome are C3 and C5, along with C3a and C3b (which are produced as the cleavage fragments of the C3 or C5 cleavage and act as anaphylatoxic agents) and intracellular receptors C3aR and C3bR, located on endosomal or lysosomal membranes. C3 and C5 can be either produced intracellularly or taken up from the external environment and transported within the cell. Intracellular activation of components does not follow classical/lectin/alternative pathway logic, for example, protease cathepsin L can cleave C3, generating C3a with no pathway activation involvement (28). This has been especially been observed in human CD4+ T cells, where in their resting state, intracellular C3a is produced, while lysosomes harboring C3aR on their surfaces. As the CD4+ T cells activate, the fast translocation of C3-associated molecules allows for an autocrine signaling resulting in cytokine production and cell differentiation (29). Disturbances in the functioning of the complosome have already been linked to some chronic diseases related to inflammation, such as arthritis, systemic lupus erythematosus (SLE), atherosclerosis, cancer and kidney pathologies (28).

## 3 The inflammasome

Innate immunity allows our bodies to defend against new pathogens, environmental irritants, and tissue damage by triggering inflammation when immune cells recognize pathogen-associated molecular patterns (PAMPs) or damage-associated molecular patterns (DAMPs) that are not normally present in the body (30). This inflammatory response is mediated in part by large multiprotein complexes called inflammasomes, which act as molecular sensors of PAMPs, DAMPs, and exogenous environmental danger signals, leading to inflammasome formation. Depending on the signal, the active inflammasome triggers caspase activation, resulting in the cleavage and maturation of the proinflammatory cytokines IL-1β and IL-18, as well as pyroptosis, a highly inflammatory form of programmed cell death (31). While this response is essential for host defense and tissue repair, persistent or dysregulated inflammasome activation promotes chronic inflammation and contributes to the pathogenesis of multiple diseases, including metabolic, cardiovascular, neurodegenerative, and autoimmune disorders (32).

This section discusses key inflammasome pathways in chronic inflammation, focusing on the well-studied NLRP3 inflammasome and the non-canonical inflammasome pathway, which are most strongly linked to complement activation, while briefly noting other inflammasomes (NLRP1, NLRC4, AIM2, Pyrin) implicated in chronic disease.

### 3.1 The NLRP3 inflammasome

The NLRP3 inflammasome, formed by theNOD-like receptor (NLR) family member NLRP3, is the most extensively studied and widely implicated inflammasome in chronic inflammatory diseases.

It is a cytosolic multiprotein complex that assembles in response to a wide range of endogenous and exogenous danger signals, including PAMPs, DAMPs, and cellular stress signals, thereby promoting inflammatory responses and preventing further tissue damage (31). Canonical NLRP3 activation requires a two-step mechanism: (i) a priming step, typically mediated by upstream pattern recognition receptors (PRR) such as Toll-like receptors (TLRs) that sense PAMPs and DAMPs and activate NF-κB signaling, which induces expression of NLRP3 and proinflammatory cytokines (33, 34); and (ii) an activation step triggered by cellular stress events such as potassium efflux, lysosomal disruption, mitochondrial dysfunction, reactive oxygen species, or extracellular ATP (35).

Upon activation, NLRP3 oligomerizes with the adaptor protein ASC (apoptosis-associated speck-like protein containing a caspase recruitment domain), leading to caspase-1 activation and cleavage of pro-IL-1β and pro-IL-18 into the active cytokines IL-1β and IL-18. Activated caspase-1 also cleaves gasdermin D (GSDMD), whose N-terminal domain forms membrane pores, triggering pyroptosis – a lytic, proinflammatory form of programmed cell death – and contributing to the rapid escalation of inflammation and the release of DAMPs, which further recruit and activate immune cells (31, 35).

While this inflammatory cascade is beneficial during acute immune responses, sustained NLRP3 activation drives chronic inflammation and tissue damage.

A hallmark of NLRP3 is its ability to detect endogenous danger signals associated with chronic disease states. In gout, the deposition of monosodium urate (MSU) crystals activates NLRP3 in joint tissues, inducing IL-1β release and acute inflammatory flares that can progress to chronic tophaceous inflammation with repeated episodes (35). The central role of NLRP3 in both acute and chronic gout is further demonstrated by the clinical efficacy of IL-1β inhibitors such as anakinra, canakinumab, and rilonacept (36, 37). In chronic respiratory diseases such as asthma and smoking-related chronic obstructive pulmonary disease (COPD), NLRP3 activation in airway cells in response to irritants and particulates has been implicated in airway inflammation and remodeling, although its precise role remains under investigation (33, 38). Similarly, periodontitis has been linked to NLRP3 activation by periodontal pathogens and gingival danger signals, potentially connecting oral inflammation with systemic disease (39). Moreover, gain-of-function mutations in NLRP3 cause cryopyrin-associated periodic syndromes (CAPS), characterized by excessive IL-1β production and systemic inflammation, and are effectively treated by IL-1 blockade (40–42).

The disease-specific roles of NLRP3 and its interplay with complement activation are discussed in detail in Section 5.

### 3.2 Non-canonical inflammasome pathway

In addition to the canonical inflammasome pathway, which requires priming for caspase-1 activation, a non-canonical inflammasome pathway involves direct activation of inflammatory caspases by cytosolic lipopolysaccharide (LPS). In mice, this pathway is mediated by caspase-11, while in humans it involves caspase-4 and caspase-5 (43). Intracellular LPS from Gram-negative bacteria binds directly to caspase-11 or caspase-4/5, inducing their oligomerization and activation independently of a NOD-like receptor (NLR). Activated caspases then cleave GSDMD, triggering pyroptosis and promoting IL-1β and IL-18 release either directly or through secondary activation of the NLRP3 inflammasome and caspase-1. Although essential for host defense against Gram-negative bacteria, persistent activation of this pathway can contribute to chronic inflammatory pathology.

While the non-canonical pathway primarily senses a single potent stimulus, LPS, usually during acute infection or endotoxemia, chronic low-level activation due to microbiome dysregulation or increased intestinal permeability can lead to sustained caspase-11 activation and chronic inflammation. In metabolic disorders, a high-fat diet induces metabolic endotoxemia, characterized by persistently elevated circulating LPS resulting from altered gut microbiota and barrier dysfunction (44, 45). In non-alcoholic fatty liver disease (NAFLD) and non-alcoholic steatohepatitis (NASH), persistent gut-derived endotoxin chronically activates caspase-11 in liver macrophages, promoting pyroptosis and IL-1β/IL-18 release, which drives disease progression (41). Caspase-11 deficiency protects mice from high-fat diet–induced NASH by reducing hepatic inflammation and pyroptotic injury. Similarly, in adipose tissue, low-level LPS sustains inflammation via caspase-11, exacerbating NLRP3-driven insulin resistance (44, 46).

### 3.3 Other inflammasomes

Several other inflammasomes contribute to chronic inflammatory diseases, although their functional connection to complement is less well defined or unknown compared to NLRP3.

The NLRP1 inflammasome is activated by pathogen-derived proteases and UV stress (47). Rare gain-of-function mutations cause the monogenic disorder NLRP1-associated autoinflammation with arthritis and dyskeratosis (NAIAD), characterized by constitutive inflammasome activation, skin hyperkeratosis, recurrent fevers, arthritis, and increased skin cancer risk (48), while common polymorphisms are associated with polygenic autoimmune diseases, including vitiligo, autoimmune Addison’s disease, type 1 diabetes, and systemic lupus erythematosus (49).

The NLRC4 inflammasome detects bacterial ligands, triggering caspase-1 activation, pyroptosis, and inflammatory responses that protect against intracellular bacteria (50, 51). Gain-of-function mutations cause constitutive IL-18 release and severe early-onset autoinflammatory diseases such as macrophage activation syndrome (MAS) and autoinflammatory infantile enterocolitis (AIFEC), while milder phenotypes, including familial cold autoinflammatory syndrome type 4 (FCAS4), present with periodic fevers, cold-induced urticaria, and arthritis, although variability in genotype–phenotype correlations has been reported (52–54).

AIM2 acts as a cytoplasmic sensor for double-stranded DNA and, when aberrant, contributes to autoimmune and autoinflammatory diseases such as psoriasis and multiple sclerosis inflammation, while loss of AIM2 activity has been associated with increased tumorigenesis (55–59). NLRP6 is predominantly expressed in the gut, where it senses microbial ligands such as lipoteichoic acid and LPS to promote IL-18 release, mucus production, and epithelial barrier integrity, with dysregulated NLRP6 function linked to dysbiosis, increased susceptibility to inflammatory bowel disease, and colitis-associated tumorigenesis. Mutations in NLRP7, which is expressed in human monocytes and macrophages, are linked to recurrent hydatidiform moles, a gestational disorder caused by dysregulated inflammation at the maternal–fetal interface, indicating a role in reproductive immune regulation (31, 60, 61).

NLRP12 primarily acts as a negative regulator of inflammation by inhibiting both canonical and noncanonical NF-κB signaling. Truncating mutations in NLRP12 result in excessive IL-1β release, increased inflammation in multiple tissues, and greater susceptibility to chronic inflammatory disorders such as colitis, inflammation-induced tumorigenesis, arthritis, neuroinflammation, and metabolic inflammation (62–65). In contrast, C1q-mediated upregulation of NLRP12 during apoptotic cell clearance suppresses caspase-1 and NLRP3 activation to maintain immune homeostasis (66).

The pyrin inflammasome detects pathogen-induced disruptions of RhoA GTPase signaling rather than directly sensing pathogens. It assembles upon RhoA inactivation to promote caspase-1–dependent IL-1β and IL-18 release (67–69). Dysregulated pyrin activity due to mutations in the MEFV gene leads to autoinflammatory diseases such as familial Mediterranean fever (FMF) and pyrin-associated autoinflammation with neutrophilic dermatosis (PAAND), which are effectively treated with IL-1–targeted therapies (70–72).

The **Table 1** summarizes key inflammasomes, their typical activation triggers, involvement in chronic inflammatory diseases, and any known links to the complement system, based on current research.

**Table 1:** An overview of key inflammasomes, their triggers, involvement in chronic inflammatory diseases, and links to the complement system.

Inflammasome	Key triggers	Chronic inflammatory diseases/conditions	Link to complement
NLRP3 (canonical; caspase-1 pathway)	Different PAMPs and DAMPs (ATP, toxins, MSU, cholesterol crystals, mitochondrial ROS, etc.). Two-step activation: priming and activation (33, 35)	Gouty arthritis, atherosclerosis, type 2 diabetes and obesity, asthma, COPD, periodontal disease, autoinflammatory syndromes (e.g. CAPS), Alzheimer’s disease, etc. (33, 35, 40, 115, 158)	Yes. Activation triggered through sub-lytic MAC deposition, C3a and C5a. Dampened through C1q (3, 66, 73, 82)
NLRP3 (non-canonical; caspase-4/5/11pathway)	Intracellular LPS from Gram-negative bacteria (43)	Contributes to chronic inflammation, e.g. liver inflammation in NAFLD/NASH; IBD; cystic fibrosis (46, 163, 167)	No specific complement receptor is known to engage this pathway.
NLRP1	Proteolytic cleavage or severe cellular stress signals (e.g. anthrax lethal toxin, UV-B radiation, certain viral proteases) (49)	Skin and autoimmune disorders, e.g. NAIAD; increased risk of vitiligo, SLE, type 1 diabetes, skin cancers, etc. (48)	Not known except for NLRP1 activation in Schwann cells through C5a binding to its receptor C5aR1, leading to IL-1β release and chronic nerve inflammation in a model of endometriosis-associated pain (177)
NLRC4	Intracellular bacterial ligands (flagellin, components of bacterial type III secretion systems) in combination with NAIP proteins (50, 51)	Protection against intracellular bacteria by inducing pyroptosis and IL-1β/IL-18 release. Mutations in NLRC4 involved in autoinflammatory diseases, presenting with MAS, enterocolitis, FCAS4 syndrome (52, 54)	Not known
AIM2	Bacterial, viral, also self dsDNA from damaged cells or mitochondria, present in the cytoplasm (55)	Overactivation linked to psoriasis, multiple sclerosis; loss of AIM2 associated with higher incidence of colorectal cancer (56, 58, 59)	Not known
NLRP6	Microbial metabolites and gut flora signals (178)	Key regulator of intestinal homeostasis. Mutations in NLRP6 linked to more severe colitis, higher susceptibility to IBD, chronic intestinal disorders (179, 180)	Not known
NLRP7	Acylated lipopeptides from microbes (31)	Dysfunctions linked to reproductive inflammation, e.g. familial biparental hydatidiform mole (60, 61)	Not known
NLRP12	Various stimuli but exact triggers are not well-defined. It attenuates inflammation by interfering with NF-κB and MAPK signaling (62)	NLRP12 mutations cause FCAS2; NLRP12 deficiency exacerbates inflammation (e.g. heightened susceptibility to colitis, colon tumorigenesis); possible link to atopic dermatitis (63, 64, 181)	Yes. C1q binding to apoptotic cells triggers an anti-inflammatory response and upregulates NLRP12, which in turn inhibits caspase-1 and the NLRP3 inflammasome (66)
Pyrin	RhoA GTPase inactivation by certain bacterial toxins and effectors (67, 68)	Recessive mutations in *MEFV* gene (pyrin) cause FMF; rarer dominant mutations in pyrin cause PAAND; also linked to HIDS (70, 182, 183)	Not known

COPD, Chronic obstructive pulmonary disease; CAPS, Cryopyrin-associated periodic syndromes; HIDS, Hyperimmunoglobulin-D syndrome; FCAS4, Familial cold autoinflammatory syndrome-4; FCAS2, Familial cold autoinflammatory syndrome-2; FMF, Familial Mediterranean fever; IBD, Inflammatory bowel disease; MAS, Macrophage activation syndrome; MSU, Monosodium urate crystals; NAFLD, Non-alcoholic fatty liver disease; NASH, Non-alcoholic steatohepatitis; NAIAD, NLRP1-associated autoinflammation with arthritis and dyskeratosis; PAAND, Pyrin-associated autoinflammation with neutrophilic dermatosis; SLE, Systemic lupus erythematosus.

## 4 Crosstalk between complement and inflammasomes: mechanism, triggers and pathological amplification

There is a growing body of evidence suggesting that the complement system and inflammasomes act together during inflammatory responses. Among the various inflammasomes, the NLRP3 inflammasome is considered the primary platform functionally linked to complement activation (73). NLRP3 responds to a wide range of danger signals, many of which can also activate the complement system indirectly by triggering other immune cells. For example, triggers such as microcrystals and extracellular ATP can activate both NLRP3 and complement pathways simultaneously (4, 74).

In recent years, much attention has been guided towards the complosome, the complement system inside the cells. Complosome activation can also lead to the initiation of the NLRP3 response, as the priming and activation of inflammasomes can also occur via complement components, which were not involved in the common three pathway activation of the complement system, but rather via an intracellular pathway, as C3a and C5a can be generated intracellularly (28). Similarly, activation of the complosome promotes the generation of mitochondrial reactive oxygen species (ROS) and metabolic reprogramming. This may lead to a lower activation threshold of the NLRP3 inflammasome and lead to caspase-1 activation and IL-1β maturation in response to secondary danger signals (75).

Additional major point of intersection is the complement MAC, which forms pores in target cell membranes, resulting in osmotic cell death. Sub-lytic assembly of the MAC on the membranes of immune cells such as macrophages and dendritic cells acts as a direct inflammasome-activating signal (73, 76). Specifically, MAC insertion can disrupt cellular ion homeostasis, causing ionic fluxes (K^+^ efflux, Ca^2+^ influx) and cellular stress, thereby initiating NLRP3 inflammasome assembly. This leads to caspase-1 activation and subsequent release of IL-1β and IL-18 (73, 76, 77). IL-1β in particular plays a critical role in several inflammatory diseases and is elevated in many conditions characterized by complement overactivation. If NLRP3 or its adaptor ASC are absent, complement activation can fail to induce the release of IL-1β and IL-18, confirming the central role of the NLRP3 inflammasome (78).

### 4.1 C5a and C3a signaling leading to inflammasome priming and activation

Although recent studies have implicated MAC, the end product of the complement cascade, as the trigger for inflammasome activation, other proteins in the complement cascade upstream of MAC can also contribute to inflammasome activation. Studies in the 1980s by Haeffner-Cavallion et al. showed that the anaphylatoxin C3a induces IL-1β production in human monocytes, indicating a functional connection between the complement system and the inflammasome before the inflammasome was even discovered (79). Since then, several studies have shown that the bioactive complement fragments C3a and C5a are important drivers of Signal 1 for NLRP3 inflammasome activation in human phagocytes and are therefore key molecular bridges linking complement activation to inflammasome responses (80–82).

The complement anaphylatoxins C5a and C3a are small peptides released during complement activation that bind to their G-protein–coupled receptors (GPCRs), C5aR1/C5aR2 and C3aR, on immune cells. They are extremely potent inflammatory mediators, regulating a wide range of immune and non-immune functions such as proinflammatory cytokine production, vasodilation, histamine release, chemoattraction, and tissue regeneration (3). By binding to GPCRs, they trigger signaling cascades such as PI3K–Akt, p38 MAPK, and calcium mobilization, ultimately leading to the activation of NF-κB and other pathways that promote inflammation and immune responses (83, 84). This can serve as a priming signal for inflammasome activation and initiates a cascade that increases the production of key components such as NLRP3 protein and pro-IL-1β, thereby priming the inflammasome. It can also directly promote a second activation signal through mechanisms such as ROS production and ionic fluxes (85).

It has been shown that C3a binding to its receptor, C3aR, enhances TLR-induced IL-1β production in monocytes by activating the ERK1/2 signaling pathway, which promotes the efflux of intracellular ATP into the extracellular space through as-yet unidentified ATP-releasing channels. The resulting increase in extracellular ATP activates the P2X7 receptor, a known trigger for NLRP3 inflammasome activation. In human monocytes, C3a amplifies LPS-induced IL-1β secretion, whereas in macrophages and dendritic cells, both C3a and LPS are required. This IL-1β production promotes T helper 17 (Th17) cell differentiation *in vitro*, and C3a generation has been associated with monocyte and Th17 infiltration in rejecting kidney transplant biopsies, highlighting a pathophysiological role for C3aR-mediated inflammasome activation in tissue inflammation (80). This study exemplifies the coordinated integration of complement signaling with TLR-driven inflammasome activation.

In addition to C3a, C5a plays a central role in inflammasome regulation in response to sterile danger signals, particularly crystalline materials. Cholesterol crystals (CC), MSU, and calcium phosphate crystals activate complement pathways, leading to C5a generation (81, 82). These crystals, which are classical NLRP3 activators through lysosomal damage and ROS production, show enhanced inflammasome activation in the presence of C5a. Samstad et al. demonstrated that CC activate both the classical and alternative complement pathways, with C5a enhancing IL-1β release and ROS generation in a complement-dependent manner, and identified CR3 as the receptor mediating CC uptake (82). Similarly, An et al. showed that MSU crystals induce C5a–C5aR1–dependent IL-1β production in whole blood via caspase-1 activation, K^+^ efflux, intracellular Ca^2+^ flux, and cathepsin B activity (86). Moreover, mitochondrial C5aR1 signaling has been linked to increased ROS generation and IL-1β gene expression, reinforcing a feed-forward loop of inflammasome activation (87).

### 4.2 Complement C1q in inflammasome activation and inhibition

Complement component C1q, the initiator of the classical pathway, acts as a dual regulator of inflammasomes, either promoting or suppressing their activation depending on context and molecular interactions. Beyond its canonical role in recognizing immune complexes, microbes, and apoptotic cells to trigger complement, C1q directly modulates immune cell signaling and inflammasome activity.

#### 4.2.1 C1q as an activator of the inflammasome

In certain pathological conditions, C1q can promote inflammasome overactivation and inflammation. For example, in rheumatoid arthritis (RA), C1q synergizes with the acute-phase protein pentraxin 3 (PTX3) to promote overactivation of the NLRP3 inflammasome and subsequent pyroptosis in monocytes (88). PTX3, which is elevated in RA, binds to C1q and enhances monocyte priming, leading to increased caspase-1 activation, GSDMD cleavage, IL-1β release, and pyroptosis. This C1q–PTX3 axis suggests a complement–pentraxin amplification loop that sustains NLRP3 activation in sterile inflammatory settings, thereby reinforcing chronic synovial inflammation (88, 89). This mechanism represents a crucial interaction between complement/pentraxin and inflammasome-mediated inflammation, serving as a potential therapeutic target for reducing persistent inflammatory cytokine release in RA.

In neurodegenerative diseases such as Alzheimer’s disease (AD), C1q and downstream complement components like C3 colocalize with amyloid plaques, leading to activation of the complement cascade, generation of anaphylatoxins and MAC, and increased microglial activation and synapse elimination (90, 91). This complement–inflammasome crosstalk creates a self-reinforcing inflammatory loop, as evidenced by improved pathology and cognition in NLRP3- or caspase-1-deficient AD mouse models (92, 93).

Similar complement–inflammasome amplification mechanisms have been proposed in other chronic inflammatory and degenerative conditions, including age-related macular degeneration (AMD) and certain forms of sterile injuries of the central nervous system (CNS) such as stroke and multiple sclerosis, where persistent C1q deposition sustains innate immune activation (94, 95). Together, these findings support the concept that C1q, while protective in the physiological clearance of debris, can under chronic stress conditions shift toward a proinflammatory role that promotes NLRP3 inflammasome activation and tissue damage.

#### 4.2.2 C1q as an inhibitor of the inflammasome

Conversely, C1q suppresses inflammasome activation during apoptotic cell clearance, efferocytosis. When C1q-opsonized apoptotic cells are engulfed by macrophages, they induce the production of type I interferons, IL-27, and IL-10, reprogramming macrophages toward an anti-inflammatory state and suppressing NLRP3 and caspase-1 activation (66). This process helps maintain immune homeostasis and prevents autoimmunity. C1q deficiency in mice and humans is linked to the development of SLE-like disease due to impaired clearance of apoptotic debris, resulting in elevated IL-1β levels and inflammation. The addition of C1q to deficient macrophages or restoration of C1q levels in patients (e.g., via plasma transfusion) can ameliorate disease and restore anti-inflammatory function, confirming its protective role against autoimmunity (66, 96).

The bidirectional effects of C1q are context- and cell-type specific. In RA monocytes, it promotes pyroptosis; in macrophages engulfing apoptotic cells, it suppresses IL-1β (88, 97). In SLE, C1q-skewed immune complexes reduce IFN-α and IL-1β in plasmacytoid dendritic cells (98). In the central nervous system, microglia produce and respond to C1q, which acts with IL-1α and TNF to induce A1 astrocytes – neurotoxic cells implicated in AD and amyotrophic lateral sclerosis (ALS). C1q deposits at synapses contribute to neurodegeneration, and its deletion reduces inflammasome-mediated neuroinflammation in AD and ALS models (99).

**Figure 1:**
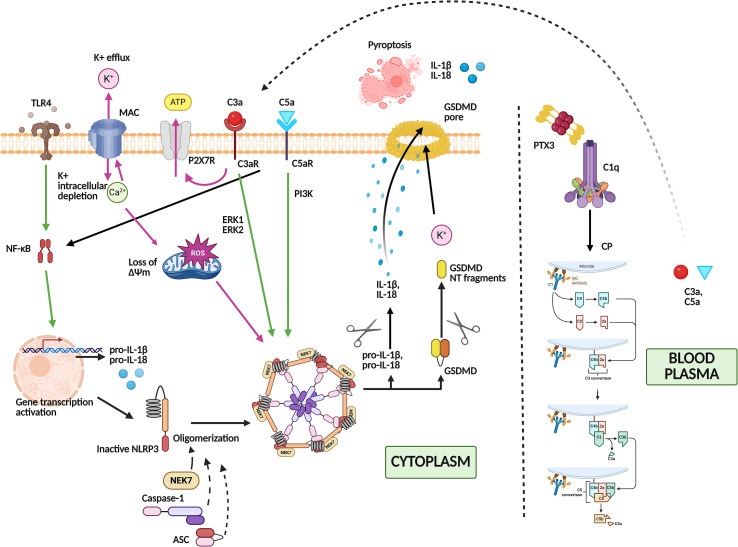
Schematic representation of the crosstalk between the complement system and NLRP3 inflammasome activation. Engagement of TLR4 triggers NF-κB signaling and induces transcription of pro-IL-1β and pro-IL-18 (Signal 1, green arrows). Binding of immobilized PTX3 to C1q promotes activation of the classical complement pathway. This interaction enhances formation of the C3 and C5 convertases, leading to increased generation of the anaphylatoxins C3a and C5a. These fragments bind to their respective receptors, C3aR and C5aR, on the cell surface, thereby enhancing monocyte priming and activating downstream signaling pathways, including NF-κB, ERK1/2, and PI3K. NF-κB activation further amplifies transcription of NLRP3 and pro-IL-1β, strengthening inflammasome priming. Formation of the MAC and stimulation of the purinergic receptor P2X7R by C3a-induced ATP release can provide Signal 2 (red arrows) for inflammasome activation through K^+^ efflux, Ca^2+^ influx, mitochondrial dysfunction (loss of mitochondrial membrane potential, ΔΨm), and increased production of ROS. The NLRP3 inflammasome assembles with the adaptor ASC and pro-caspase-1, resulting in caspase-1 activation. Active caspase-1 processes pro-IL-1β and pro-IL-18 into their mature forms and cleaves GSDMD, whose N-terminal fragment forms membrane pores, leading to pyroptotic cell death and release of IL-1β and IL-18. TLR4, Toll-like receptor 4; PTX3, Pentraxin 3; NLRP3, NOD, Nucleotide-binding oligomerization domain; LRR, leucine-rich repeat; PYD, pyrin domain-containing protein 3; MAC, Membrane attack complex; P2X7R, P2X7 Receptor; ROS, Reactive oxygen species; ASC, Apoptosis-associated speck-like protein containing a caspase recruitment domain; GSDMD, Gasdermin D; GSDMD NT fragments, Gasdermin D N-terminal fragments; NEK7, NIMA-related kinase 7; CP, Classical pathway.

A schematic representation of the crosstalk between the NLRP3 inflammasome and the complement system is illustrated in **Figure 1**.

### 4.3 Shared activating signals

The complement system and inflammasomes are often activated by common triggers, as both detect signs of infection or tissue damage. These shared stimuli ensure that complement and inflammasome pathways are co-activated in many inflammatory scenarios, reinforcing each other’s responses (3). Below are summarized key overlapping triggers.

#### 4.3.1 Pathogen-associated molecular patterns

Many microbial components can activate complement (e.g., bacterial cell walls activate the alternative complement pathway, immune complexes activate the classical pathway) while simultaneously providing signals for inflammasomes or related pathways (3, 10). For example, Gram-negative bacteria trigger complement and also release LPS, which binds to the TLR4 complex on immune cells, providing a “priming” signal (Signal 1) for the NLRP3 inflammasome (43). Bacterial toxins and secreted factors, such as pore-forming toxins or extracellular ATP, can directly activate NLRP3 (Signal 2). Similar to bacterial pore-forming toxins, the complement MAC also acts as a pore-forming complex on target membranes, causing ion flux and triggering the assembly and activation of the NLRP3 inflammasome (23). Flagellin, the primary protein component of bacterial flagella used for motility, is detected inside the macrophage cytosol by NAIP proteins. Upon binding flagellin, NAIPs recruit the NLRC4 protein, leading to the assembly of the NLRC4 inflammasome complex. In addition, the complement system opsonizes these bacteria, enhancing the efficiency of phagocytosis (100). In viral infections, complement may be activated via the lectin pathway, triggered by the binding of mannose-binding lectin (MBL) and ficolins to specific carbohydrate structures (glycans) found on the surface of pathogens, including viruses and virally infected cells (101). Additionally, viral RNA and DNA can trigger RLRs or AIM2 inflammasomes (102).

Thus, during infections, it is common to see parallel activation of complement and inflammasomes, each recognizing different aspects of the pathogen.

#### 4.3.2 Damage-associated molecular patterns

Sterile tissue injury triggers the release of various host molecules known as DAMPs, which activate both the complement system and inflammasome pathways. Extracellular ATP, released from damaged cells, activates inflammasomes via the P2X7 receptor, triggering K^+^ efflux – a critical step in the assembly and activation of the inflammasome complex that leads to the production of inflammatory cytokines (103). The release of ATP also indicates cell lysis, which can initiate the complement cascade, which is spontaneously activated on exposed intracellular contents or membranes lacking regulatory proteins (104). ROS and oxidative stress, often elevated in injured tissues, serve as key signals for activating the NLRP3 inflammasome (105). Additionally, oxidative modifications of proteins and lipids can create neoantigens that may activate the complement system (106). Uric acid crystals in gout and cholesterol crystals in atherosclerotic plaques also act as DAMPs, triggering inflammation by engaging both the NLRP3 inflammasome and the complement system (107).

#### 4.3.3 Mitochondrial damage and cellular stress

Mitochondria, due to their bacterial origins, contain specific molecules such as mitochondrial DNA (mtDNA), N-formyl peptides, and cardiolipin, which, when released, act as DAMPs. Injured cells or ischemic tissue often release mitochondrial contents into the extracellular space. The immune system can recognize this mitochondrial debris as foreign, potentially activating components of the immune response, such as the complement system, specifically the lectin or alternative pathways. These same mitochondrial DAMPs are also recognized as potent activators of NLRP3 inflammasomes within nearby immune cells, driving significant inflammatory responses (108, 109). For example, in conditions such as myocardial infarction or stroke, cell necrosis leads to complement activation on cell debris and simultaneously triggers inflammasomes in infiltrating macrophages due to DAMP release (110). ROS generated from damaged mitochondria activate the NLRP3 inflammasome and can amplify complement activation by promoting an inflammatory environment (4). In essence, mitochondrial dysfunction acts as a central trigger in a vicious cycle of inflammation: the MAC of the complement system may worsen mitochondrial injury, while mitochondrial ROS subsequently promote inflammasome activation. This self-perpetuating feedback loop drives chronic inflammatory responses in various diseases (4).

#### 4.3.4 Crystalline and particulate stimuli

In addition to urate and cholesterol crystals, other particulates such as silica dust, asbestos fibers, amyloid aggregates, and environmental particles can simultaneously activate both the NLRP3 inflammasome and the complement system. Inhaled silica and asbestos are phagocytosed by macrophages and lung epithelial cells, leading to lysosomal damage and the release of lysosomal contents into the cytoplasm, which triggers the assembly and activation of the NLRP3 inflammasome complex (111). These particulates also activate the complement system through distinct mechanisms. Asbestos fibers (e.g., crocidolite, chrysotile) primarily engage the alternative pathway by directly binding factor B and properdin, forming the C3 convertase and generating C3a and C5a, which drive chronic lung inflammation. In contrast, silica particles can bypass traditional pathways by catalyzing hydroxyl radical–mediated oxidation of C5, which is then cleaved by kallikrein to produce C5a. Some studies suggest that silica may also engage the alternative pathway, but this appears to be context dependent. Both types of particles ultimately lead to C5a-driven inflammatory cell recruitment, contributing to diseases such as asbestosis and silicosis (112, 113).

**Figure 2:**
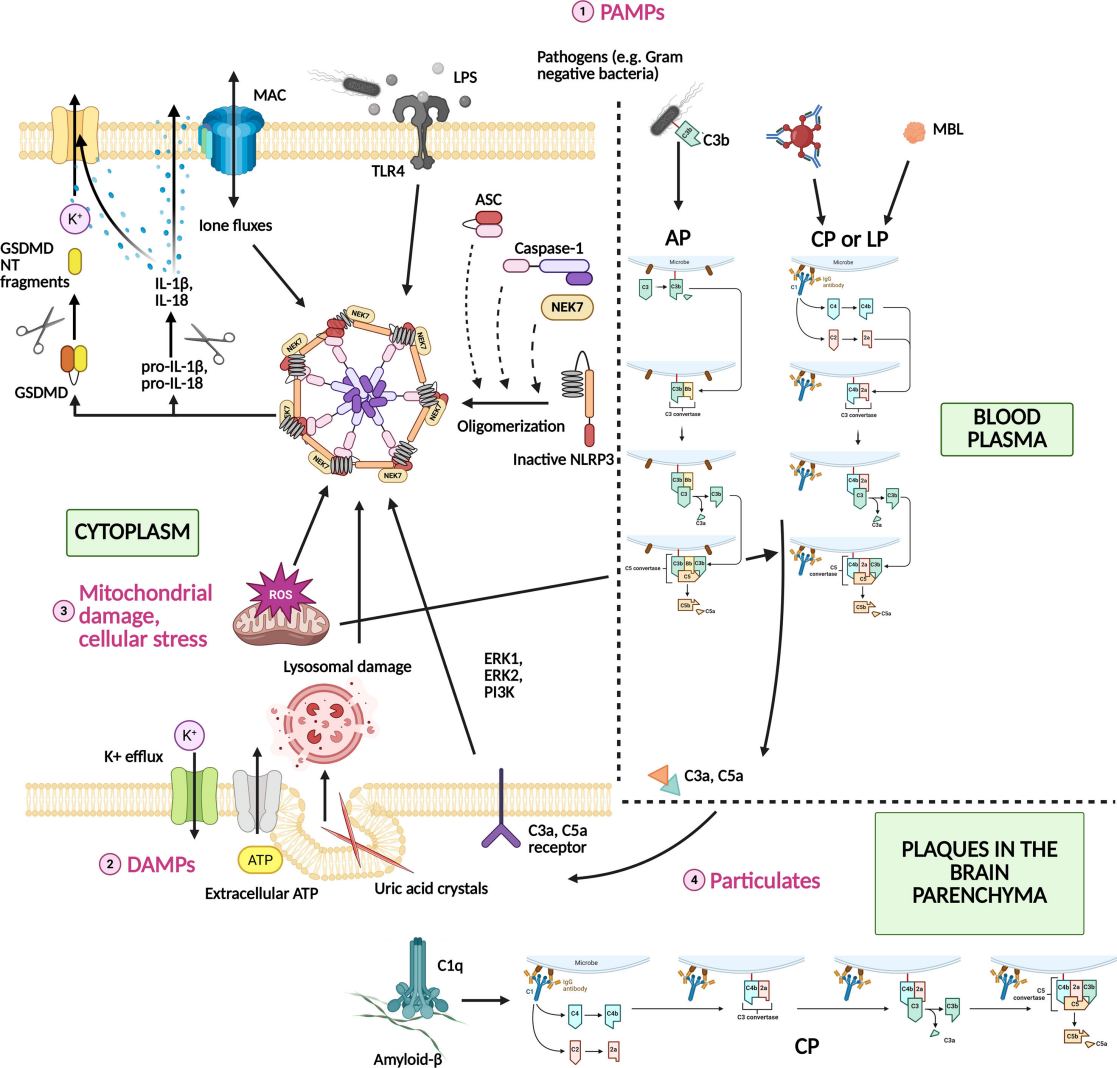
Shared activation signals triggering both the complement system and inflammasome pathways. The complement system and inflammasomes are often co-activated by common danger signals from pathogens or tissue damage. (1) PAMPs, such as components of Gram-negative bacteria (LPS), activate complement pathways in blood plasma while simultaneously engaging PRRs on immune cells (e.g., TLR4). The complement MAC, like microbial pore-forming toxins, induces ion fluxes that promote inflammasome assembly. (2) DAMPs released during sterile tissue injury, including extracellular ATP, promote K^+^ efflux and activate inflammasomes via purinergic receptors, while also triggering complement activation on damaged or unprotected membranes. (3) Mitochondrial damage and cellular stress generate ROS and release mitochondrial DAMPs, which are potent activators of the NLRP3 inflammasome and can also amplify complement activation, reinforcing inflammatory signaling. (4) Crystalline and particulate stimuli, such as uric acid crystals, silica, asbestos, and amyloid-β aggregates, cause lysosomal damage after phagocytosis, leading to inflammasome activation. These particulates also directly (by binding to C1q) or indirectly activate the complement cascade, generating pro-inflammatory mediators such as C3a and C5a. PAMPs, Pathogen-associated molecular patterns; LPS, Lipopolysaccharide; PRRs, Pattern-recognition receptors; TLR4, Toll-like receptor 4; MAC, Membrane attack complex; DAMPs, Damage-associated molecular patterns; ROS, Reactive oxygen species; NLRP3, NOD, Nucleotide-binding oligomerization domain, LRR, leucine-rich repeat; PYD, pyrin domain -containing protein 3; GSDMD, Gasdermin D; GSDMD NT fragments, Gasdermin D N-terminal fragments; ASC, Apoptosis-associated speck-like protein containing a caspase recruitment domain; NEK7, NIMA-related kinase 7; CP, Classical pathway; AP, Alternative pathway; LP, Lectin pathway.

**Figure 2** provides a schematic overview of shared activation signals for the inflammasome and complement system.

### 4.4 Feedback amplification loops in inflammatory diseases

#### 4.4.1 Complement upregulation and inflammasome activation

Complement activation amplifies inflammasome signaling through bidirectional crosstalk. Complement-derived anaphylatoxins provide both priming (Signal 1) and activation (Signal 2) cues for the NLRP3 inflammasome. C5a binding to C5aR1 on myeloid cells induces NF-κB–dependent transcription of IL-1β and NLRP3, while also promoting mitochondrial stress and reactive oxygen species production via PI3K-dependent pathways, thereby lowering the threshold for inflammasome activation (114). In parallel, C3a signaling through C3aR promotes the release of intracellular ATP, which activates the P2X7 receptor and induces the potassium efflux required for NLRP3 assembly (78). Additionally, sublytic MAC formation can induce ion fluxes and mitochondrial perturbation, further contributing to inflammasome activation and caspase-1–dependent maturation of IL-1β and IL-18 (73, 76).

Complement signals can have both enhancing and suppressive effects on inflammasome activity depending on the inflammatory context. In atherosclerosis, cholesterol crystals activate complement via C1q, and downstream generation of C5a works with TNF-α to prime macrophages for NLRP3 activation (115). Similarly, in gout, C5aR1 signaling increases NLRP3-dependent IL-1β release through ROS production, promoting neutrophil-driven inflammation, while pharmacological blockade of C5aR reduces this response (116, 117). In contrast to these proinflammatory settings, complement can also limit inflammasome activation: C1q binding to apoptotic cell debris restricts caspase-1 cleavage and IL-1β production during efferocytosis, likely by inducing IL-10 and the NF-κB suppressor NLRP12 (4). Loss of this regulatory pathway may be especially relevant in SLE, where C1q deficiency or consumption is common and may worsen NLRP3-, IL-1β-, and IL-18–driven inflammation (4).

Complement–inflammasome feedback forms a self-perpetuating cycle. Complement fragments C3a and C5a are potent proinflammatory peptides that recruit and activate leukocytes such as neutrophils and monocytes. C5a, in particular, enhances IL-1β production by priming the NLRP3 inflammasome, acting as a second signal or cooperating with other stimuli like TNF-α (117). Proinflammatory cytokines, including IL-1β and TNF-α, increase complement synthesis in various tissue cells. For example, studies using human intestinal epithelial Caco-2 cells confirm that IL-1β stimulates C3 mRNA and protein production, a process further enhanced by IFN-γ (7, 8). This creates an inflammatory feed-forward loop in which complement activation products promote inflammasome-driven cytokine release, and those cytokines, in turn, increase local complement synthesis, fueling further inflammation and tissue damage. During an inflammatory flare (e.g., in sepsis or arthritis), IL-1β and other cytokines upregulate complement levels, ensuring abundant substrate for ongoing complement activation (6). This self-reinforcing loop sustains chronic inflammation in diseases such as RA, where immune complexes activate complement, and C5a and C3a fragments recruit IL-1β–producing leukocytes, amplifying synovial inflammation and tissue damage. C5a and its receptor, C5aR, are indeed found at elevated levels in the synovial fluid and tissue of RA patients and contribute to leukocyte influx, while IL-1β induces production of other cytokines (IL-6, IL-8), proteases, and adhesion molecules in the RA synovium, driving chronic inflammation, pannus formation, cartilage erosion, and bone destruction (117, 118).

#### 4.4.2 IL-1β autocrine inflammatory loop

Once IL-1β is produced and secreted, it can act on the same cell (autocrine) or neighboring cells (paracrine) by binding to the interleukin-1 receptor type 1 (IL-1R1) and triggering MyD88-dependent NF-κB and MAPK signaling pathways. Activated transcription factors (NF-κB and AP-1 from MAPK) translocate to the nucleus, where they induce the expression of various inflammatory genes, including the gene for IL-1β itself. Thus, IL-1β induces expression of its own gene, creating a positive feedback loop that amplifies the initial inflammatory signal and sustains the inflammatory response (119, 120). Both IL-1α and IL-1β can upregulate their own expression, thereby sustaining the IL-1 response in inflamed tissues. This loop is reinforced by IL-1β’s ability to stimulate other inflammatory mediators (IL-6, TNF-α, chemokines), which can further enhance IL-1β synthesis or stability. NF-κB activation also primes NLRP3, further supporting ongoing IL-1β maturation (121). IL-1β’s autocrine loop greatly amplifies local inflammation.

IL-1β also upregulates other inflammatory pathways, including the complement system. NF-κB activation by IL-1 leads to increased synthesis of acute-phase proteins in the liver, driven largely by IL-6, and local production of complement components. Experiments in human epithelial cells show that IL-1β can markedly increase C3 synthesis at the transcriptional level, which is further boosted by IFN-γ co-exposure. This synergy establishes a cytokine-complement circuit, where IL-1β boosts complement, and complement fragments (e.g., C5a) prime further IL-1β release, sustaining chronic inflammation (122, 123).

#### 4.4.3 IL-18 and the IFN-γ axis

IL-18 is another key inflammasome-derived cytokine that operates in a feedback network connecting innate and adaptive immunity. Its primary function is to induce IFN-γ production from T helper 1 (Th1) cells and natural killer (NK) cells in the presence of IL-12 or other cofactors, forming a positive feedback loop: IL-18 promotes IFN-γ release by lymphocytes, and IFN-γ in turn activates macrophages and other cells to amplify inflammation and boost IL-1β/IL-18 production. However, IFN-γ also induces the production of IL-18 binding protein (IL-18BP), a natural inhibitor that provides a crucial negative feedback mechanism to balance IL-18 activity and prevent excessive inflammation (124, 125).

IFN-γ has multiple proinflammatory effects. It enhances macrophage microbicidal activity, PRR and MHC expression, and inflammasome priming by inducing transcription factors such as IRF1 that heighten responsiveness to secondary stimuli (126).

In chronic immune activation, this IL-18–IFN-γ axis can become pathogenic. In MAS, hemophagocytic lymphohistiocytosis (HLH), and Still’s disease, excess IL-18 drives uncontrolled IFN-γ production and immune activation, causing a cytokine storm. Blocking IL-18 or IFN-γ alleviates these conditions (127, 128). In SLE, elevated IL-18 correlates with disease severity, particularly nephritis, and fuels IFN-γ responses that activate mesangial cells and macrophages. Although SLE patients produce IL-18BP, a natural IFN-γ–induced IL-18 inhibitor, it often fails to fully neutralize IL-18, leading to persistent inflammation (129, 130).

The IL-18–IFN-γ axis also intersects with complement and IL-1 loops. IFN-γ boosts C3 synthesis and primes cells to generate inflammasome signals such as ROS and pro-IL-1β. IL-18 itself can act on macrophages in an autocrine manner to induce inflammatory genes (131).

#### 4.4.4 Leukocyte recruitment and tissue damage

IL-1β, along with IL-1α – which serves as the primary initiator of sterile inflammation and is released from necrotic or dying cells as a preformed alarmin – drives neutrophil recruitment. IL-1β subsequently amplifies this response by inducing endothelial cells to express key adhesion molecules (e.g., ICAM-1, E-selectin) and chemokines such as CXCL5 and CXCL8 (132, 133). The resulting influx of neutrophils and monocytes leads to the release of proteases, oxidants, and neutrophil extracellular traps (NETs), which cause localized tissue damage and the release of intracellular DAMPs. Extracellular ATP and oxidized mitochondrial DNA typically activate the NLRP3 inflammasome via K^+^ efflux, while cytosolic dsDNA is directly sensed by the AIM2 inflammasome (134). This feedback loop sustains the inflammatory cycle and perpetuates tissue injury.

Neutrophil enzymes such as elastase and cathepsin G can also activate complement by cleaving C3 and C5 into chemotactic C3a and, more potently, C5a, further enhancing leukocyte recruitment and forming a reinforcing loop (135, 136). Inflammasome cytokines recruit leukocytes, which amplify cytokine production through complement and further inflammasome activation. In autoinflammatory diseases such as CAPS, driven by constant NLRP3 inflammasome activity, patients show elevated IL-1β and IL-18 levels, along with increased complement proteins in blood, reflecting IL-1–induced hepatic complement synthesis and complement’s contribution to inflammation (137). IL-1 blockade in CAPS markedly reduces systemic inflammation, underscoring the central role of IL-1–driven loops in sustaining complement-mediated inflammation (138).

## 5 Implication of complement and inflammasome in disease states

It was long believed that the main role of complement and inflammasome was only defense against pathogens. Today we know that both complement and inflammasome have many other roles in health and pathology. In recent years major advances have been made in the field of psychiatry as well (139, 140). In the following subsections we discuss examples of complement and inflammasome involvement in some of the chronic inflammation associated pathologies.

### 5.1 Alzheimer’s disease

Chronic inflammatory diseases of the CNS, such as AD, a neurodegenerative disorder with a significant inflammatory component, are strongly linked to NLRP3 inflammasome activation and complement signaling. In AD, aggregated amyloid-β (Aβ) peptides act as DAMPs that trigger microglial uptake, leading to lysosomal rupture, cathepsin B release, and activation of the NLRP3 inflammasome (35). This results in the secretion of proinflammatory cytokines IL-1β and IL-18, as well as pyroptotic signaling, which amplify neuroinflammation and contribute to synaptic dysfunction and neuronal loss (35, 141, 142). Beyond C1q recognition, Aβ fibrils broadly activate the complement system, as both C1q and downstream components such as C3 colocalize with amyloid plaques, leading to full activation of the complement cascade and generation of inflammatory mediators, including anaphylatoxins C3a and C5a and the MAC. These complement effectors further promote microglial activation, synapse elimination, and neurodegenerative processes characteristic of AD pathology (91, 143). This complement–inflammasome interplay forms a self-perpetuating loop, enhancing IL-1β production and neurodegeneration. Consistently, NLRP3 or caspase-1 deficiency in AD mouse models leads to reduced plaque burden and improved cognitive performance (144, 145). While NLRP3 plays a central role, other inflammasomes may also contribute to CNS inflammation in diseases such as Parkinson’s and multiple sclerosis (35). Overall, the convergence of complement activation and inflammasome signaling in AD exemplifies how innate immune pathways synergize to drive chronic neuroinflammation and progressive tissue injury.

Moreover, IL-18, key inflammasome-derived cytokine, has been implicated in chronic neuroinflammation. In AD, IL-18 from microglia reduces amyloid clearance and promotes neuroinflammation by stimulating IFN-γ and other mediators in glia, collectively contributing to cognitive decline. Although IL-1β has been the main focus in AD, IL-18 levels are also elevated in AD patients’ brains and cerebrospinal fluid in some studies, suggesting it participates in the neuroinflammatory milieu (146, 147).

### 5.2 Atherosclerosis

In atherosclerosis, a chronic inflammatory cardiovascular disease, NLRP3 is activated by endogenous lipid-derived crystals and metabolic stress. Cholesterol crystals that accumulate in atherosclerotic plaques are recognized as DAMPs, triggering the NLRP3 inflammasome in plaque macrophages. Oxidized LDL and other metabolic byproducts can also activate NLRP3, leading to the release of IL-1β and IL-18, which drive vascular inflammation and plaque progression (115). NLRP3-driven inflammation thus contributes to lesion development and instability in atherosclerosis; therefore, NLRP3 inhibition is being explored as a therapeutic strategy in cardiovascular disease (148, 149). In parallel, the complement system plays a crucial role in sustaining vascular inflammation throughout all stages of atherosclerosis. Complement activation products, including C3a and C5a, are detected in large amounts in atherosclerotic lesions and promote the recruitment and retention of monocytes and neutrophils within the arterial wall. C5a–C5aR1 signaling enhances NLRP3 activation, ROS production, and proinflammatory cytokine release, and by that amplifying local inflammation. In addition, sublytic C5b-9 deposition induces endothelial dysfunction and smooth muscle cell activation, contributing to plaque progression and instability (150, 151).

### 5.3 Gout and RA

In gout, deposition of MSU crystals in the synovium, synovial bursa, cartilage, and other joint tissues activates the NLRP3 inflammasome, leading to IL-1β release and acute arthritis flares. Recurrent episodes drive chronic tophaceous inflammation (35). IL-1β released in response to MSU crystals stimulates nearby cells to produce additional IL-1 and chemokines, which recruit neutrophils and monocytes that further amplify IL-1β production, fueling a self-reinforcing inflammatory loop (117, 152). The critical role of NLRP3 in both acute and chronic gout is underscored by the clinical efficacy of IL-1β inhibitors such as anakinra, canakinumab, and rilonacept (36, 37). In parallel, MSU crystals activate the classical pathway of the complement system. In the presence of CRP, this activation is enhanced even more. C5-converatse that forms on the surface of the MSU crystals leads to C5a generation, which promotes neutrophil recruitment and amplifies inflammatory cytokine production within the joint. Complement activation synergizes with NLRP3 inflammasome signaling by enhancing myeloid cell activation and sustaining IL-1β–driven inflammation, contributing to both acute gout flares and the transition to chronic state (153). In RA, IL-1β induces synovial macrophages and fibroblasts to upregulate matrix metalloproteinases and additional IL-1, thereby perpetuating joint damage and inflammation (154). Although IL-1β also stimulates production of its natural antagonist IL-1Ra, this compensatory mechanism is often insufficient to control inflammation in chronic disease settings (155). Many patients with rheumatoid arthritis have detectable autoantibodies such as anti-citrullinated protein autoantibodies (ACPA) and rheumatoid factors. Immune complex–mediated complement activation generates C3a and C5a within the synovium, which cooperate with IL-1β signaling to perpetuate leukocyte recruitment, synovial hyperplasia, and joint destruction (156, 157).

### 5.4 Type 2 diabetes

Metabolic disorders also illustrate chronic NLRP3 activation. In type 2 diabetes (T2D) and obesity, persistent metabolic signals such as hyperglycemia and accumulation of islet amyloid polypeptides (IAPP) in the pancreatic islets in T2D act as NLRP3 triggers and lead to IL-1β secretion (158). Chronic NLRP3 activation in islets and insulin-responsive tissues contributes to insulin resistance, as IL-1β impairs insulin signaling and promotes β-cell dysfunction. In obesity, adipose tissue macrophages show increased NLRP3 activity, since excess nutrients such as saturated fatty acids and elevated urate levels (from high-purine diets) serve as continuous NLRP3 stimuli, causing release of IL-1β and IL-18 that maintain low-grade inflammation. Visceral adipose tissue in obese individuals has higher NLRP3 and IL-1β expression than subcutaneous fat, correlating with systemic insulin resistance (159). In experimental models, NLRP3 deficiency or pharmacologic inhibition can improve insulin sensitivity and glucose tolerance, highlighting NLRP3’s role as a driver of chronic metabolic inflammation (160, 161). In addition to inflammasome activation, the complement system contributes to chronic metabolic inflammation in T2D and obesity. Upregulation of complement components, especially C3, is a marker of insulin resistance. Both circulating and locally produced complement components, particularly C3, C3a, and C5a, are elevated in obese and insulin-resistant individuals. This promotes recruitment and activation of myeloid cells in adipose tissue. C3aR and C5aR1 signaling contributes to low-grade inflammation by enhancing cytokine production, subsequentially enhancing liver C3 production and further impairing insulin signaling in adipocytes and macrophages, reinforcing metabolic dysfunction (162).

### 5.5 Intestinal inflammation

Chronic intestinal inflammatory conditions, such as inflammatory bowel disease (IBD), can also involve the non-canonical inflammasome pathway. In IBD, the non-canonical inflammasome plays a dual role, acting as both a driver and a potential protector of inflammation (163). It is activated by cytoplasmic LPS, detected when bacteria or their components enter the cell cytosol due to breaches in the mucosal barrier and changes in gut flora (164). This can lead to sustained activation of caspase-4/5 or -11 in intestinal macrophages and epithelial cells, driving pyroptosis, amplifying mucosal inflammation, and promoting tissue damage. Increased caspase-5 expression and pyroptotic cell death have been observed in colon biopsies from patients with active IBD, linking the non-canonical pathway to chronic intestinal inflammation, although NLRP3 and other inflammasomes also play roles (165, 166). Chronic infections by Gram-negative bacteria or repeated exposure to their LPS in tissues such as the lungs can also engage caspase-4 or -11, triggering pyroptosis and promoting the release of proinflammatory cytokines. For example, in cystic fibrosis or bronchiectasis patients colonized with *Pseudomonas aeruginosa*, persistent LPS exposure may continually trigger caspase-4 or -11–mediated IL-1β release in the airways, contributing to neutrophilic inflammation and tissue damage over time (167). In contrast, multiple studies in mouse models of colitis have shown that the caspase-11 inflammasome can have a protective role in intestinal inflammation, with caspase-11–deficient mice experiencing much more severe colitis. Moreover, IFN-γ–mediated caspase-11 activity contributes to maintaining the intestinal epithelial barrier and gut homeostasis, partly by ensuring adequate IL-18 production, which promotes the proliferation and repair of intestinal epithelial cells and prevents excessive inflammatory cell death (163, 168). Complement activation also plays an important role in shaping intestinal inflammation. The intestines provide a crucial physical and immunological barrier. Intestinal epithelial cells can produce complement components. Activation of the complement system in the intestines can be beneficial when it is low-level, local, and controlled. In contrast, when local production of C3 and C5 within the intestinal mucosa leads to persistent generation of C3a and C5a, it can promote recruitment and activation of myeloid cells and contribute to epithelial barrier dysfunction. Complement signaling can synergy with inflammasome by amplifying inflammatory cytokine release and sustaining tissue-damaging immune responses during chronic disease. At the same time, excessive or dysregulated complement activation may add to mucosal injury (169).

## 6 Discussion

In summary, there is significant overlap in the triggers for complement and inflammasomes. Pathogens, danger signals such as ATP, oxidative stress, and crystals do not exclusively activate one system; they often initiate cascades involving both. This overlap ensures a robust inflammatory response, with complement providing immediate opsonization, chemoattraction, and direct cell lysis, and inflammasomes generating potent IL-1β and IL-18 signals that recruit and activate leukocytes. The result is a coordinated innate immune defense which, when chronic or dysregulated, also contributes to the progression of several inflammatory diseases.

### 6.1 Therapeutic implications and future directions

As the complement system and inflammasome are involved in chronic inflammation, there is a growing need for better, integrated diagnostics and therapy. A broad overview of most used and researched drug are highlighted in **Table 2**. Notably, the anticomplement therapy has seen great success, with anti-C5 monoclonal antibodies markedly improving patient status in renal pathologies. As these monoclonal antibodies prevent the cleavage of C5 into C5a, their anti-inflammatory effects could be useful for the treatment of other inflammatory conditions. Additional anticomplement therapies focus on inhibiting initiation, amplification of effects of complement (170). Currently, to the best of our knowledge, there are no approved drugs that would directly target inflammasomes. There are however drugs that influence the effects of the activation of the inflammasome (such as IL-1 signaling) (171). While therapeutic blockade of IL-1 using agents such as anakinra (an IL-1 receptor antagonist) is a recognized treatment strategy for RA and related autoinflammatory conditions, specific C5aR antagonists (e.g., PMX53) or anti-C5 antibodies (e.g., eculizumab) have shown efficacy in animal models of arthritis but have not consistently demonstrated significant clinical improvement or reduction of synovial inflammation in human RA patients (117, 118, 172).When drug mechanism interacts with a large, complex biological pathways as complement system and inflammasome, certain consequence cannot be avoided as the effects can be observed throughout the pathway. Such example are eculizumab, an anti-C5 monoclonal antibody and pegcetacoplan, a pegylated C3 inhibitor. By inhibiting the complement, patients using such treatments have an increased risk for meningococcal disease, namely *Neisseria meningitidis*, as well as *Streptococcus pneumoniae* and *Hemophilus influenzae* type B infections, therefore advised to use antibiotic prophylaxis and additional vaccinations (173). Additional disadvantages are the limited availability of treatment and its very high cost. As an examples, eculizumab treatment is estimated to cost around half a million yearly per patient (174).

**Table 2:** Complement system and inflammasome associated drugs that have entered in clinical trials/are in use.

Drug	Possible disease treatment use	Target/mechanism of action	Phase of clinical development	References
Eculizumab	PNS aHUS NMOSD	Anti-C5 monoclonal antibody	FDA and EMA approved	(184)
Ravulizumab	PNS aHUS MG NMOSD	Anti-C5 monoclonal antibody	FDA and EMA approved	(185)
Avacopan	ANCA associated vasculitis	C5aR1 antagonist	FDA and EMA approved	(186)
Pegcetacoplan	PNS AMD C3G IC-MPGN	Pegylated C3 inhibitor	FDA and EMA approved	(187)
Iptacopan	PNS C3G	Factor B inhibitor	FDA approved EMA approved as orphan medicine	(188)
Narsoplimab	TA-TMA IgA nephropathy	Anti-MASP-2 monoclonal antibody	FDA approved EMA under evaluation	(189)
Sutimlimab	CAD	Anti-C1s monoclonal antibody	FDA and EMA approved	(190)
MCC950	heart and kidney disease, injury, inflammatory bowel disease, and neurological conditions	NLRP3 inhibitor	Stopped during a Phase 2 rheumatoid arthritis (RA) trial due to liver toxicity	(171)
Dapansutrile	gout, joint pain and COVID-19	NLRP3 inhibitor	Phase 2 Efficacy Trial	(191)
VTX2735	Recurrent Pericarditis CAPS	NLRP3 inhibitor	Phase 2a	(192, 193)
Anakinra	RA CAPS COVID-19	IL-1 receptor antagonist	FDA and EMA approved	(194)
Canakinumab	CAPS FMF SJIA	anti-IL-1β monoclonal antibody	FDA and EMA approved	(195)

ANCA, Anti-neutrophil cytoplasmic (auto)antibody; aHUS, Atypical hemolytic uremic syndrome; AMD, Age-related macular degeneration; C3G, C3 glomerulopathy; CAPS, Cryopyrin-associated periodic syndromes; CAD, Cold Agglutinin Disease; FMF, Familial Mediterranean Fever; IC-MPGN, Primary immune complex membranoproliferative glomerulonephritis; MG, Myasthenia gravis; NMOSD, Neuromyelitis optica spectrum disorder; PNS, Paroxysmal nocturnal hemoglobinuria; SJIA, Systemic Juvenile Idiopathic Arthritis; TA-TMA, Hematopoietic stem cell transplant-associated thrombotic microangiopathy.

Recent clinical data further emphasize the translational relevance of complement–inflammasome crosstalk. In the early post-infusion phase of chimeric antigen receptor T (CAR-T) cell therapy, synchronized activation of the complement cascade and the NLRP3 inflammasome has been observed, contributing to systemic inflammation, pyroptosis, and cytokine release syndrome (CRS). Complement activation products appear to enhance inflammasome priming and IL-1β release, while inflammasome-driven cytokines further amplify complement activation, creating a self-propagating inflammatory circuit. These findings underscore the importance of targeting complement and/or NLRP3 pathways to mitigate hyperinflammatory toxicities during CAR-T immunotherapy and improve patient outcomes (175).

These observations expand the therapeutic landscape beyond traditional autoimmune and metabolic diseases by providing a strong mechanistic rationale for targeting both complement and inflammasome pathways in inflammatory and immune-mediated conditions. Persistent activation of complement effectors (such as C5a, C3a, and MAC) and NLRP3-driven IL-1β and IL-18 release creates self-amplifying inflammatory circuits that sustain tissue injury in diseases including RA, SLE, atherosclerosis, neurodegeneration, and metabolic syndrome (4, 77). Therapeutic strategies that interrupt these loops – using C5 inhibitors, C5aR1 antagonists, NLRP3 inhibitors, caspase-1 blockade, or IL-1–targeted therapies – are therefore mechanistically justified, as they directly disrupt both upstream priming signals and downstream cytokine amplification (3, 176). Importantly, dual or combinatorial targeting strategies may offer advantages over single-pathway inhibition. Because complement activation provides both priming (Signal 1) and activation (Signal 2) signals to the NLRP3 inflammasome, and inflammasome-derived cytokines further enhance complement production, simultaneous modulation of both systems may more effectively disrupt this feed-forward loop (3, 73). Sequential or combination approaches – such as pairing C5aR1 antagonists with NLRP3 inhibitors or IL-1 blockade – could reduce residual inflammatory signaling, limit compensatory pathway activation, and improve clinical control in refractory or severe disease phenotypes. These concepts are especially relevant in translational settings such as CAR-T–associated CRS, where coordinated complement and NLRP3 inflammasome activation drive systemic inflammation (175). Integrated intervention strategies targeting complement and inflammasome signaling could help reduce hyperinflammatory toxicities without compromising antitumor efficacy. Future studies should focus on identifying predictive biomarkers of complement–inflammasome overactivation, stratifying high-risk patients, and clinically evaluating rational combination therapies in both chronic inflammatory diseases and immunotherapy-associated toxicities.

## 7 Conclusions

In summary, the complex crosstalk between the complement system and inflammasomes plays a crucial role in sustaining chronic inflammation in various diseases. Although both pathways are central to innate immunity, they have long been overlooked in routine diagnostics and therapy. Recent advances, however, are beginning to recognize this interface as a critical and actionable target. The potential for dual or synergistic targeting of complement and inflammasome components offers promising new therapeutic strategies, especially in conditions where traditional treatments fail to resolve inflammation. Further mechanistic and translational studies are needed to define context-specific interventions and identify reliable biomarkers. Emerging tools such as spatial omics, patient-derived organoids, and machine learning-based disease stratification may help unravel the complexity of complement–inflammasome networks and guide precision immunomodulatory therapies in the near future.
